# Physical activity and weight status of children in Germany: cross-sectional results from the MoMo Wave 3 (2018–2020)

**DOI:** 10.1007/s00431-025-06183-9

**Published:** 2025-05-24

**Authors:** Elena Brehm, Lara Tschuschke, Leon Klos, Alexander Burchartz, Carmen Volk, Anke Hanssen-Doose, Darko Jekauc, Claudia Niessner, Alexander Woll

**Affiliations:** 1 Institute of Sports and Sports Science, Karlsruhe Institute of Technology, Engler-Bunte-Ring 15, 76131 Karlsruhe, Germany; 2 Institute of Movement and Sport, Karlsruhe University of Education, Bismarckstr. 10, 76133 Karlsruhe, Germany

**Keywords:** Body mass index, Sports club physical activity, Leisure time physical activity, Physical activity recommendations

## Abstract

Childhood physical activity (PA) plays a critical role in preventing obesity and supporting overall health. This study investigates the prevalence of weight status categories and their association with organized and unorganized PA among 6- to 17-year-old children in Germany using MoMo Wave 3 (2018–2020). Cross-sectional data from MoMo Wave 3 (2018–2020) were analyzed to examine associations between weight status and PA. A total of 1983 participants (age: 11.6 ± 3.4 years, 52.3% male) completed a questionnaire and had anthropometric measurements taken, including height and weight. Statistical comparisons between normal-weight children and those classified as underweight or overweight based on their body mass index focused on their participation in organized and unorganized PA. Linear and logistic regressions were used for statistical analysis. Among the participants, 3.0% were severely underweight, 5.8% were underweight, 7.2% were overweight, and 4.6% were obese. Participants classified as underweight (*ꞵ* = − .084; *p* < .001) and overweight (*ꞵ* = − .045; *p* = .042) spent less time in organized PA compared to normal weights. Overweight participants were less likely to participate in unorganized PA (OR = .64, *p* = .003). No significant differences were observed in the duration of unorganized PA among those who participated, regardless of weight status.

*Conclusion*: The prevalence of overweight and obesity has remained consistently high in recent years. Tailored interventions should address the specific barriers faced by both underweight and overweight children to enhance their participation in PA and improve health outcomes across diverse groups.

**Table:** 

**What is Known:** • *Children with overweight or obesity are less physically active than their normal-weight peers, underweight children are often as physically active as those with normal weight*.
**What is New:** • *Children with overweight and underweight show less organized physical activities compared to their normal-weight peers*. • *Although overweight children engaged less frequently in unorganized physical activities, those who did participated for a similar duration as normal-weight children*.

**What is Known:**

• *Children with overweight or obesity are less physically active than their normal-weight peers, underweight children are often as physically active as those with normal weight*.

**What is New:**

• *Children with overweight and underweight show less organized physical activities compared to their normal-weight peers*.

• *Although overweight children engaged less frequently in unorganized physical activities, those who did participated for a similar duration as normal-weight children*.

## Introduction

Childhood overweight and obesity pose significant health risks, both physically and psychologically [1, 2], often persisting into adulthood [3]. In Germany, 15.4% of children aged 3–17 are classified as overweight or obese [4], a rate that has remained consistently high in recent years [3], reflecting broader trends observed across Europe [5]. In addition, underweight in children also represents a serious health concern, particularly during key developmental stages. In industrialized countries, being underweight is often linked to body image concerns or clinical conditions [6], contributing to the risk of eating disorders, especially among girls [7], and social stigmatization among boys [8]. In Germany, 2.4% of children aged 3–17 are classified as severe underweight, while 5.2% are classified as underweight based on the most recent representative KiGGS data (2014–2017) on children’s weight status in Germany [4]. To effectively monitor the prevalence trends and inform targeted prevention efforts, updated data are essential.

Although overweight and obesity occur due to an imbalance between energy intake and energy expenditure, it is typically a multifactorial condition driven by obesogenic environments [9]. Physical activity (PA) is a pivotal factor in preventing and managing childhood overweight and obesity, as emphasized by the World Health Organization (WHO) [10]. Stodden et al. [11] postulated a reciprocal relationship between weight status and PA in children, suggesting that overweight children spend less time with moderate to vigorous PA per day than normal-weight children [12]. Moreover, overweight children are less likely to meet the WHO’s 2010 PA guidelines [12–16]. The relationship between PA and underweight status is less clear, with some studies reporting no differences in PA levels between underweight and normal-weight children [13, 14], while Kantanista et al. [17] observed significantly lower PA levels among 14- to 16-year-old underweight boys in Poland.

Barriers to PA participation are particularly pronounced for children with either overweight or underweight status [18, 19], which may contribute to differences in PA domains. Only a few studies specifically analyzed the weight status in different PA domains [17, 20–22]. Kreuser et al. [21] found no differences in sports club membership between normal-weight and overweight children in Germany. However, while 13.4% of the children with normal weight notified engagement in organized individual or team sports or other organized training more than three times per week, none of the overweight children did so. In an Australian cohort of 9- to 16-year-olds, obese children engaged in significantly less free play than normal-weight children, whereas overweight and underweight did not differ from those with normal weight [22]. Similar patterns have been observed in Poland [17]. These variations may reflect country-specific socio-economic factors or cultural attitudes toward PA and weight status, which merit further investigation. Therefore, a deeper understanding of the association between weight status and various PA domains is relevant for identifying opportunities to promote PA among underweight and overweight children in Germany.

The economic burden of childhood obesity and underweight on healthcare systems is substantial, with significant costs related to medical treatment and productivity loss [23]. As part of its commitment to the Sustainable Development Goals, Germany has pledged to combat noncommunicable diseases, encompassing conditions like obesity, reflecting a broader dedication to global sustainability efforts [24]. Nevertheless, evidence on the association between weight status and PA remains limited and warrants further research. Continuous monitoring of weight status and the association with PA is necessary for understanding their correlation and tailoring interventions for vulnerable populations, aligning with national and global public health goals. Consistent and representative cross-sectional studies serve as a foundation for devising effective strategies to address obesity and associated health concerns within these particular groups [25, 26].

Building on this context, the primary purpose of this study is to (a) determine the prevalence of weight status categories from cross-sectional data of the MoMo Wave 3 (2018–2020) and (b) to explore the potential influence of weight status on PA among 6- to 17-year-old children in Germany. Specifically, the study investigates differences in organized and unorganized PA across various weight status categories. We hypothesize that children who were overweight or underweight spend less time within different settings of PA than normal weight children. The findings of this study are intended to guide policymakers, with an emphasis on implementing preventive strategies to enhance PA, while addressing the needs of vulnerable groups.

## Methods

### Study design and participants

The database of this investigation is formed by the MoMo study, a nationwide study aimed to assess physical fitness, PA, and health in children. It was developed as an in-depth module of the German Health Interview and Examination Survey for children (KiGGS) [27] from the Robert Koch-Institute with a cohort-sequence design [28]. A nationwide, stratified, multistage sample was drawn during each period of measurement (described as “waves”) to maximize representativeness [29]. A systematic sample of 167 primary sampling units was drawn from a list of German communities, stratified by level of urbanization and geographic region. Selection probability was proportional to the number of residents under 18 [30]. Each wave incorporates both new participants and a longitudinal sample. For sampling of MoMo Wave 3, a stratified sample was selected by random from the 167 municipalities of the former study waves. In the second step, an age-stratified random sample of children and adolescents between 4 and 17 years was selected from official population registers. A total of 140 residents per sampling unit was selected. Due to the contact restrictions and lockdowns imposed in March 2020 because of the outbreak of the COVID-19 pandemic, MoMo Wave 3 had to be interrupted prematurely. Nevertheless, data from *N* = 3391 participants aged 4–17 years from 114 locations in Germany were collected successfully between July 2018 and March 2020. Parents and adolescents were invited by letter to examination rooms at central locations in close proximity to participant’s homes in municipalities where the study was conducted. For minors, parents gave their written consent. For participants under the age of 16, the presence of a legal guardian was mandatory. Participants had to fill out questionnaires on-site on laptops about their PA behavior. Anthropometric data and motor performance were measured by trained staff. Finally, participants completed an interview regarding their health behavior. For this study, we used cross-sectional data from MoMo Wave 3 participants. Inclusion criteria were an age between 6 and 17 years and complete information on sex, age, body mass index (BMI), as well as organized and unorganized PA. A positive approval of the ethics committee of Karlsruhe Institute of Technology (KIT) dated September 23, 2014, is available for the study. The STROBE statement guided the reporting of the study [31].

### Measurement

#### Weight status and body mass index

BMI was calculated based on measurements of body height in cm (*Seca 213 stadiometer*) and body weight in kg (*Seca body scale 813 robusta*)*.* Using a German reference sample, BMI percentiles were calculated according to Kromeyer-Hauschild et al. [32], taking age and sex into account. Participants were classified as severely underweight if their BMI was below the 3rd percentile, underweight between the 3rd and 10^th^ percentile, normal weight between the 10^th^ and 90^th^ percentile, overweight above the 90^th^ percentile, and obese above the 97^th^ percentile.

#### PA assessment

PA was assessed using the MoMo Physical Activity Questionnaire [33]. The questionnaire has good test–retest reliability: *ICC* = 0.68 [34]. For organized PA, participants were asked about the minutes per week they spent on curricular and extracurricular sports activities at school (e.g., extracurricular sports activities: “How long do you spend on this/these [extracurricular] sports activity/activities per week?”), as well as the minutes per week and months per year they spent on club sports activities (e.g., club sports: “How long do you usually spend doing this sport per week [in minutes] (excluding travel, changing clothes, and showering)?”). Participants could enter up to four different types of club sports activities. The minutes per week for each type of club sports activity were multiplied by a monthly factor (e.g., to account for seasonal activities), calculated as the number of months the activity was performed divided by 12. All minutes for each type of activity were then summed up. To account for school holidays, the total minutes of curricular and extracurricular sports were adjusted by multiplying them with a correction factor of 8.5 divided by 12. An index for organized PA, reflecting minutes per week, was then formed by summing the total minutes for curricular, extracurricular, and club sports activities. For unorganized PA, participants were asked about the minutes per week and months per year they spent on unorganized sports activities (e.g., unorganized sports activities: “How long do you usually spend doing this sport per week [in minutes] (excluding travel, changing clothes, and showering)?”). Participants could enter up to four different types of unorganized sports activities. The minutes per week for each type of activity were multiplied by a monthly factor, calculated as the number of months the activity was performed divided by 12 months. All minutes for each type of activity were then summed up, reflecting minutes per week for unorganized PA.

### Statistical analysis

The analysis was carried out using IBM SPSS Statistics 29.0.0.0 (241). Descriptive data were analyzed to calculate and classify BMI. Age, height, and weight are presented in mean ± standard deviation (SD). For BMI description, prevalence in % and 95% confidence intervals (CI) was determined for descriptive statistics.

Due to small sample sizes, BMI categories were combined for regression analysis as follows: “Severely underweight” and “underweight” were combined into “underweight,” and “overweight” and “obese” were combined into “overweight.” Based on theoretical model of Welk [35], all regression analyses were controlled for age group and sex, and normal weight was set as a reference group. Sensitive analyses showed that excluding participants with PA data further than 3 standard deviations from the mean did not alter the results. The outliers were retained in the dataset because they could be plausibly explained within the context of the study.

A linear regression was calculated to assess the relation between organized PA (min/week) and weight status. Since only 814 participants reported unorganized PA, logistic regression was first applied to assess the relation between weight status and unorganized PA participation (0 min/week vs. > 0 min/week). A linear regression was calculated for those that reported at least 1 min per week of unorganized PA. Nagelkerke’s *R*^2^ [36] was calculated to assess model fit for logistic regression. In linear regression, standardized beta-coefficients (*ꞵ)* and *p*-values were reported. Statistical significance for all tests was set to *α* = 0.05.

## Results

### Study population

**Fig. 1:**
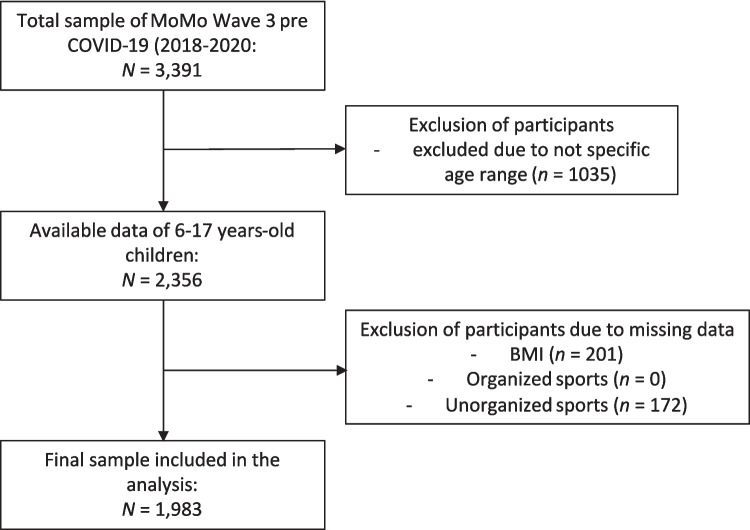
Flowchart of the study

After data cleaning and exclusion outside the specified age range (58.5% of the original 3391), the analysis focused on a final sample of *N* = 1983 participants (11.6 ± 3.4 years, 52.3% male). Figure 1 illustrates the stepwise exclusion process. Table 1 presents the prevalence of weight status categories in the overall sample of 6- to 17-year-old children. Prevalence for overweight was higher in the older age groups compared to younger ones, but not for obesity. In terms of sex, prevalence for overweight was higher in boys, and prevalence for obesity was higher in girls. For severely underweight, girls (3.5%, 95% CI 2.3–4.7) showed a slightly higher prevalence compared to boys (2.6%, 95% CI 1.6–3.6).

**Table 1:** Descriptive characteristics of sex, age, height, weight, and weight status according to Kromeyer-Hauschild et al. [32] from cross-sectional data of MoMo Wave 3 (2018–2020)

		6–10 years		11–13 years		14–17 years		Overall (6–17 years)		
		Boys (*n* = 495)	Girls (*n* = 433)	Boys (*n* = 253)	Girls (*n* = 240)	Boys (*n* = 289)	Girls (*n* = 273)	Boys (*n* = 1037)	Girls (*n* = 946)	Overall (*N* = 1983)
Sex	%	53.3	46.7	51.3	48.7	51.4	48.6	52.3	47.7	100
Age [years]	M (± SD)	8.4 (± 1.5)	8.5 (± 1.5)	12.5 (± 0.9)	12.5 (± 0.8)	15.8 (± 1.2)	15.9 (± 1.1)	11.5 (± 3.4)	11.7 (± 3.4)	11.6 (± 3.4)
Height [cm]	M (± SD)	132.4 (± 10.7)	132.8 (± 10.9)	157.3 (± 9.9)	156.4 (± 8.0)	175.3 (± 8.3)	166.8 (± 6.2)	150.5 (± 20.9)	148.6 (± 17.5)	149.6 (± 19.4)
Weight [kg]	M (± SD)	29.2 (± 7.4)	29.9 (± 8.3)	48.0 (± 12.5)	47.0 (± 10.2)	65.8 (± 12.5)	60.1 (± 10.9)	44.0 (± 18.7)	42.9 (± 16.1)	43.5 (± 17.5)
Weight status										
Severely underweight	% [95% CI]	2.6 [1.2–4.0]	3.7 [1.9–5.5]	2.4 [0.5–4.3]	4.2 [1.7–6.7]	2.8 [0.9–4.7]	2.6 [0.7–4.5]	2.6 [1.6–3.6]	3.5 [2.3–4.7]	3.0 [2.2–3.8]
Underweight	% [95% CI]	6.3 [4.2–8.4]	5.5 [3.4–7.6]	6.3 [3.3–9.3]	5.8 [2.8–8.8]	4.8 [2.3–7.3]	6.2 [3.3–9.1]	5.9 [4.5–7.3]	5.8 [4.3–7.3]	5.8 [4.8–6.8]
Normal weight	% [95% CI]	82.2 [78.8–85.6]	79.2 [75.4–83.0]	77.5 [72.4–82.6]	78.8 [73.6–84.0]	78.9 [74.2–83.6]	77.3 [72.3–82.3]	80.1 [77.7–82.5]	78.5 [75.9–81.1]	79.4 [77.6–81.2]
Overweight	% [95% CI]	6.1 [4.0–8.2]	6.0 [3.8–8.2]	8.3 [4.9–11.7]	6.7 [3.5–9.9]	9.0 [5.7–12.3]	8.4 [5.1–11.7]	7.4 [5.8–9.0]	6.9 [5.3–8.5]	7.2 [6.1–8.3]
Obesity	% [95% CI]	2.8 [1.3–4.3]	5.5 [3.4–7.6]	5.5 [2.7–8.3]	4.6 [1.9–7.3]	4.5 [2.1–6.9]	5.5 [2.8–8.2]	4.0 [2.8–5.2]	5.3 [3.9–6.7]	4.6 [3.7–5.5]

*M *mean, *SD *standard deviation, *95% CI *95% confidence interval

### Organized PA and weight status

**Fig. 2:**
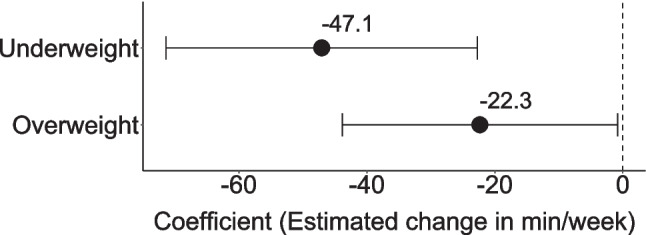
Estimated regression coefficients (*b*) and 95% confidence intervals from linear regression model examining the association between organized physical activity and weight status, ontrolled for sex and age group, based on cross-sectional data from MoMo Wave 3 (2018–2020), reference= normal weight children

Descriptive statistics on time spent in organized PA are shown in Table 2. Children classified as underweight (*ꞵ* = − 0.084; *p* < 0.001) and overweight (*ꞵ* = − 0.045; *p* = 0.042) participated in less organized PA than normal weight children (Fig. 2).

**Table 2:** Results of the linear and logistic regression analysis examining the association between organized/unorganized physical activity and weight status controlled for sex and age group from cross-sectional data of the MoMo Wave 3 (2018–2020)

	Organized physical activity^1^			Unorganized physical activity^1^				
	Linear regression model (min/week) (*n* = 1983)			Logistic regression model (yes/no) (*n* = 1983)		Linear regression model (min/week) (*n* = 817)		
Intercept	258.0			− 0.396		145.7		
	*b*	95% CI	*ꞵ*	OR	95% CI	b	95% CI	*ꞵ*
Sex (ref. boys)								
Girls	− 37.3	[− 51.1; − 23.5]	− 0.117*	1.25*	[1.04; 1.50]	− 35.2	[− 54.1; − 16.3]	− 0.124*
Age group (ref. 6–10 years)								
11–13 years	43.0	[25.9; 60.1]	0.117*	1.39*	[1.10; 1.73]	34.7	[10.4; 58.9]	0.105*
14–17 years	31.3	[15.9; 47.7]	0.089*	2.21*	[1.78; 2.74]	77.2	[55.3; 99.1]	0.261*
Weight status (ref. normal weight)^2^								
Underweight	− 47.1	[− 71.4; − 22.7]	− 0.084*	0.85	[0.61; 1.17]	2.6	[− 31.8; 36.9]	0.005
Overweight	− 22.3	[− 43.9; − 0.8]	− 0.045*	0.64*	[0.47; 0.85]	− 11.1	[− 43.5; 21.3]	− 0.023
Model fit	0.036 (0.033)^3^			0.044^4^		0.071 (0.066)^3^		

*OR *odds ratios, *95% CI *95% confidence interval, r*ef*. reference; *p < 0.05

^1^Dependent variable

^2^Weight status according to Kromeyer-Hauschild et al. [32]

^3^*R*^2^ (adjusted *R*^2^)

^4^Nagelkerkes *R*

### Unorganized PA and weight status

**Fig. 3:**
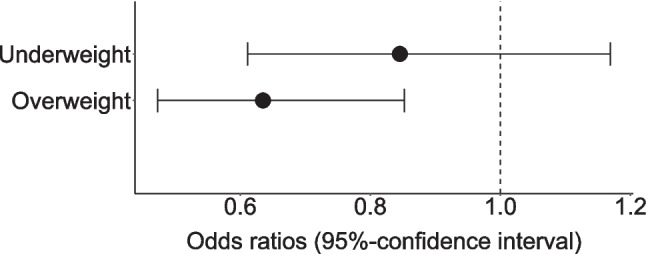
Odds ratios (OR) and 95% confidence intervals from a logistic regression model examining the association between unorganized physical activity and weight status, controlled for sex and age group, based on cross-sectional data from MoMo Wave 3 (2018–2020), reference = normal-weight children

**Fig. 4:**
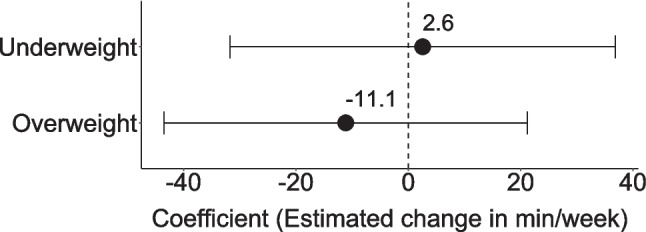
Estimated regression coefficients (*b*) and 95% confidence intervals examining the association between unorganized physical activity and weight status, controlled for sex and age group, based on cross-sectional data from MoMo Wave 3 (2018–2020), reference = normal-weight children

A total of 41.2% (*n* = 817) of the children engaged in unorganized PA. The results of the logistic regression indicated that children with underweight did not differ significantly from normal-weight children (OR = 0.85, *p* = 0.310) (Fig. 3). Overweight children differed significantly from those with normal-weight (OR = 0.64, *p* = 0.003), indicating that overweight children had 36% lower odds of engaging in unorganized PA compared to their normal-weight peers. When considering the duration of only children who engaged in unorganized PA, no significant differences were found between different weights (Fig. 4).

## Discussion

This study aimed to examine the prevalence of weight status categories among children aged 6 to 17 years in Germany, as part of MoMo Wave 3 (2018–2020). In addition, the study aimed to explore the influence of weight status on organized and unorganized PA. Normal weight children engaged in more organized PA than underweight and overweight peers. Additionally, overweight children were less likely to participate in any kind of unorganized PA. However, those participating in unorganized PA did not differ in duration based on weight status.

Compared to KiGGS Wave 2 (2014–2017), this study found lower rates of overweight (7.2% vs. 9.5%) and obesity (4.6% vs. 5.9%) [4]. Notably, solely prevalence of overweight was higher in older age groups, whereas in KiGGS Wave 2, the prevalence of obesity also increased. The discrepancy may be partly attributed to differences in age groups studied, as KiGGS Wave 2 included children as young as 3 years, potentially affecting prevalence comparisons [4]. Since MoMo emphasizes motor performance testing, children with overweight or lower motivation for sports may have been underrepresented, potentially leading to an underestimation of overweight prevalence. The lack of weighting to adjust for non-response or sample deviations may have biased the findings [37]. Weighting was not feasible due to the early termination of data collection during the COVID-19 pandemic. Prevalence of underweight in this study was similar to or slightly higher than in other European countries when considering severely underweight and underweight together [4, 17, 38].

### Organized PA and weight status

The findings highlight the significant, albeit small, influence of weight status on organized PA. Consistent with prior studies, overweight children participated less frequently in organized PA compared to their normal-weight peers [17, 21, 39]. No significant differences emerged for underweight children, consistent with previous studies [17]. In particular, children from lower socio-economic backgrounds tend to have higher rates of overweight and obesity [40], possibly due to limited awareness or financial constraints within their families, resulting in lower participation in sports clubs [41]. Additionally, it is plausible that overweight children avoid club sports due to feelings of shame, fear, or ridicule [8]. Previous research by Kantanista and Osiński (2014) has also shown that overweight children participate less in physical education classes than their normal-weight peers, whereas underweight children show no significant differences in participation [17]. Compared to our results, the differences could be explained by extracurricular activities, suggesting a need to consider socioeconomic factors more deeply. As children who engage in organized PA demonstrate higher vigorous PA levels compared to those with similar PA levels but without sports club membership [42], the role of organized PA remains crucial in terms of health-enhancing effects.

### Unorganized PA and weight status

Regarding unorganized PA, differences were observed only among overweight children, who participated less frequently than their normal-weight peers. This aligns with findings from a study with Canadian adolescents, but only for participants with obesity [43]. Interestingly, overweight participants in our study did not spend less time with unorganized PA than normal-weight children once they participated. This may suggest that while overweight children face barriers to initial participation, once engaged, they may exhibit similar PA levels to their normal-weight peers. This supports the idea that informal settings and peer support may reduce participation barriers [44]. According to potential barriers of PA due to weight status [18, 19], the results may indicate that these barriers relate to the domain of unorganized PA for overweight children, but not for those with underweight. While there is an overall decrease in unorganized PA [45], it is not clear whether underweight and overweight children are affected in the same way or whether normal-weight children have now moved closer to these groups in terms of their PA. Engagement in unorganized PA is associated with higher habitual PA and participation in organized PA [20], which underlines the importance of unorganized PA. In accordance with these results, Schmidt et al. [45] emphasized the fundamental role of schools, sports clubs, and unorganized PA during leisure time for promoting PA among young individuals. However, these findings must be interpreted with caution, as self-reported data may be prone to bias. Overweight children might underreport their PA levels due to social desirability, possibly hiding larger differences in unorganized PA [46].

## Strength and limitations

The primary strength of the study lies in its nationwide sample with almost 2000 children, coupled with extensive efforts to collect representative data from 167 sample points across Germany. Data collection in the field was conducted by trained staff, ensuring high methodological standards. Due to the premature cancellation of data collection due to the COVID-19 pandemic, it was not possible to test children from all planned sampling points. A validated questionnaire tailored to participants across various age groups was used, ensuring ease of completion and reliability [34]. An additional consideration is the absence of data on biological maturity, which could have been beneficial to control for in the analysis given its potential influence on PA [47]. Our separate analysis of organized and unorganized PA proves to be a significant strength, given our focus on the overall relevance of PA. While positive outcomes of PA are independent of its organizational structure, our data show no correlation between the two concepts (*r* = 0.001, *p* = 0.982) [48]. Other activity domains, such as active transport or outdoor play, were not assessed, which may underestimate total PA [45]. Despite time availability being a constraint for both organized and unorganized sports, the distinction is analytically valuable. This approach allowed us to identify marked differences between the two domains, providing deeper insights into their respective impacts. Finally, the cross-sectional design limits the ability to infer causality between weight status and PA levels, necessitating longitudinal studies to establish temporal relationships.

## Conclusion

This study offers important insights into the relationship between weight status and PA among 6- to 17-year-old children in Germany. Our findings reveal that the prevalence of overweight and obesity remains high, albeit slightly lower than in KiGGS Wave 2. In contrast, the proportion of underweight children is relatively small. A key observation is that both underweight and overweight children participate less in organized PA compared to their normal-weight peers. To better support these groups, school-based physical education should emphasize motivational aspects as well as opportunities to experience new forms of PA. As schools can reach all children, they play a central role in fostering long-term healthy behaviors. Collaboration between schools and sports clubs, along with targeted programs (e.g., sports classes for overweight children) in sports club settings, can help reduce participation barriers and foster long-term involvement in PA. This highlights the importance of consistent participation in organized sports, as it not only supports regular PA but may also help maintain fitness levels in the long-term benefits that are often not achieved through unstructured activity alone [49].

## Data Availability

No datasets were generated or analysed during the current study.

## Abbreviations

*BMI*: Body mass index
*PA*: Physical activity
*WHO*: World Health Organization
